# Load Capacity of Screw Anchor Installed in Concrete Substrate Reinforced with Steel Fibers Depending on Fiber Content

**DOI:** 10.3390/ma17051105

**Published:** 2024-02-28

**Authors:** Kazimierz Konieczny, Daniel Dudek, Alfred Kukiełka

**Affiliations:** Building Research Institute (ITB), ul. Filtrowa 1, 00-611 Warsaw, Poland; k.konieczny@itb.pl (K.K.); a.kukielka@itb.pl (A.K.)

**Keywords:** concrete, fiber reinforcement, screw anchor, mechanical properties, pull-out test, destructive load, pull-out strength, non-cracked concrete substrate

## Abstract

Pull-out strength tests conducted on screw anchors in uncracked concrete substrates of the C25/30 class are presented in this article. The destructive force for anchor–concrete fasting was tested, and in the next step, the average pull-out strengths of screw anchors in concrete substrates with and without the addition of steel fiber were determined. Currently, the pull-out strengths of anchors in fiber-reinforced concrete substrates are defined as for unreinforced concrete substrates. Therefore, pull-out tests were performed for screw anchors in fiber-reinforced concrete substrates. Fiber contents of 10, 20, 30, and 50 kg/m^3^ were used. An increase in the load capacity of screw anchors in a fiber-reinforced concrete substrate was demonstrated in a pull-out test compared to base samples without fibers. The coefficient related to the actual fastening behavior of a screw anchor in the fiber-reinforced concrete substrate was determined. It was assumed that a coefficient of 13.10 should be adopted. This was the lowest value obtained for the load capacity in this study for screw anchors in a fiber-reinforced concrete substrate.

## 1. Introduction

Each structure should be designed such that it fulfils its tasks in terms of usability, load-bearing capacity, and stability throughout its intended period of use without any significant reduction in its usefulness or excessive unforeseen maintenance costs. The development of civilization and its surprising pace require the use of various construction technologies. In a building’s structure, fastener elements play an important role in addition to purely structural elements. Anchors are used to enable quick and safe fastening of various types of structural and nonstructural building elements [1]. Thus, there are two types of anchors: anchors of a construction nature are used to connect elements of the structure of a facility or heavy elements of equipment (beams, columns, walls, stair supports, ventilated façade structures, drywall structures, windows, heavy chandeliers, furniture, etc.), and nonstructural anchors are used when additional light elements are installed (skirting boards, wall lamps, pictures, etc.).

Anchors are exposed to various factors during their installation and operation, such as the type and nature of the load transferred, the condition and strength of the substrate, and the temperature at which the anchors are installed and used. However, they are most often used in concrete substrates. This is consistent with the assessment of fasteners based on the EOTA (European Organisation for Technical Approvals) [2] and Eurocode 2 [3]. However, many researchers have tested the strength of fasteners depending on various conditions, such as the type of concrete, a non-cracked or cracked substrate [4,5,6,7,8], its carbonation [9,10,11,12,13], or additional impacts, including static and dynamic loads [14]. Ruopp et al. [15] investigated large anchor plates under shear loading in steel–concrete connections. However, Luo et al. [16] analyzed the connections of steel plates with concrete using anchors and adhesives in terms of shear performance. Woyciechowski et al. [13] examined the load-bearing capacity of steel fixing anchors depending on the condition of the concrete substrate, which was degraded as a result of carbonation, water absorption, and freezing.

Moreover, according to the EOTA [2] and Eurocode 2 [3], fastenings made in fiber-reinforced concrete elements were designed and implemented as typical concrete substrates. This does not correspond to the actual operating conditions of fasteners, which are often installed in elements made of fiber-reinforced concrete [10,17,18,19,20,21,22,23,24]. An example is fiber-reinforced concrete floors [25,26,27]. Fiber-reinforced concrete is resistant to cracking and crack propagation (by bridging the fibers over the crack) [28,29]. Moreover, it is characterized by higher flexural strength, resistance to impact and fatigue, and good durability [11]. Fibers can improve the stiffness and durability of reinforced concrete and prestressed structures [12]. Owing to these properties, fiber-reinforced concrete is used in the construction of buildings, bridges, tunnels, and high-strength surfaces [18]. The most widely used fiber-reinforced concrete is concrete reinforced with steel fibers [13,30,31,32,33,34]. However, only a few studies have been conducted on anchorage in fiber-reinforced concrete [35,36,37,38]. The load-bearing capacity of anchors anchored in a substrate made of steel fiber-reinforced concrete has been shown to increase with the fiber content [36,37]. Lee et al. [36] obtained a linear correlation between load-bearing capacity and fiber content. Bokor et al. [37] obtained increases in load-bearing capacity of 22% and 37%, respectively, for steel fibers in the amounts of 30 kg/m^3^ and 50 kg/m^3^. However, Gesoglu et al. [38] found that the addition of steel fibers had no significant effect on the pull-out strength of anchors, but there was a reduction in concrete damage compared to plain concrete. Dudek et al. [35] analyzed the effect of the steel fiber content on the maximum pull-out force of M10 expansion anchors installed in concrete substrates (non-cracked and cracked with a crack width of 0.30 mm) reinforced with fibers. Two concrete classes (C20/25 and C50/60) and three steel fiber contents (15 kg/m^3^, 30 kg/m^3^, and 50 kg/m^3^) were used. The study presented in this article is a continuation of this research, the goal of which is to change the technical assessment procedure and the design system for the use of steel anchors in a fiber-reinforced concrete substrate and not in a concrete substrate as before. This study used a different type of anchor: steel screw anchors. This study aimed to assess the effect of reinforcement on the load capacity of screw anchors installed in concrete substrates (non-cracked) reinforced with fibers at different depths of anchoring.

## 2. Materials

### 2.1. Steel Anchors

**Figure 1:**
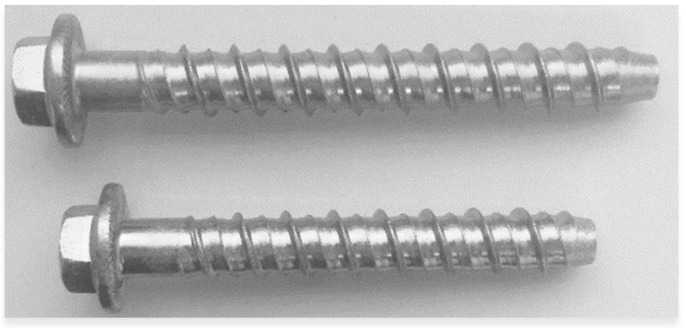
TFE14120 (**bottom**) and TFE14140 (**top**) steel screw anchors [photo taken by authors].

This study used two types of screw anchors, TFE14120 and TFE14140 (Técnicas Expansivas S.L., Logroño (La Rioja), Spain), as shown in Figure 1.

Table 1 and Table 2 present the material and installation parameters of the anchors used, respectively, which are provided in accordance with the guidelines. The first connector, with a length of 120 mm, was screwed into the concrete to depths of 65 and 75 mm, while the second connector, with a length of 140 mm, was screwed into the concrete to depths of 85, 95, and 105 mm in holes 10 mm deeper (see Table 2). The holes were drilled using a drill with a diameter of 14 mm according to [39].

**Table 1:** The material parameters of the screw anchors (see Figure 2).

No.	Type of Screw Anchor	Length L [mm]	Diameter d [mm]	Nut Diameter S_w_ [mm]
1	TFE14120	120	17	19
2	TFE14140	140	17	19

### 2.2. Concrete Substrate

The concrete substrate for the load capacity tests was made of a C25/30 concrete mixture in accordance with the EAD (European Assessment Document) [2] and the Eurocode standard [3] for steel anchors, as shown in Table 3.

**Figure 3:**
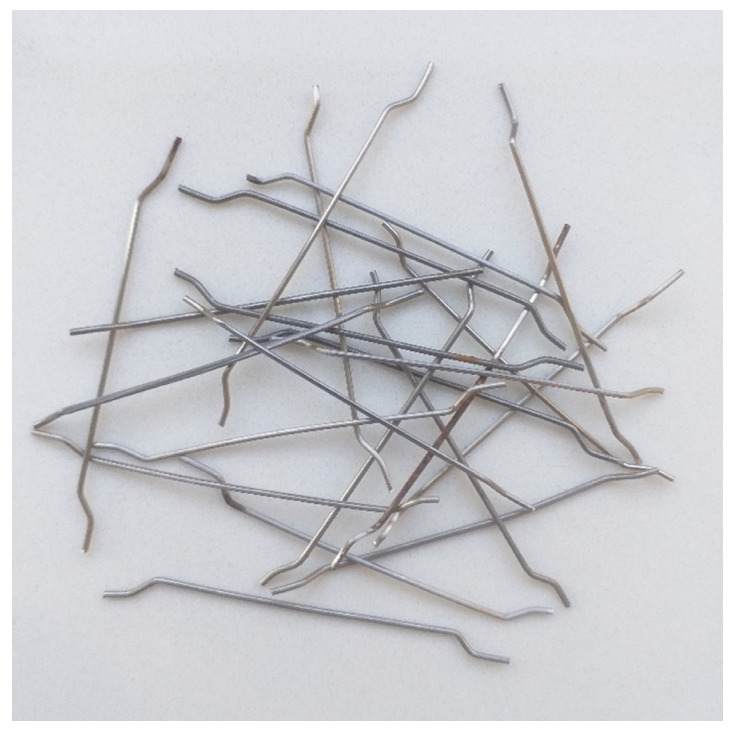
Steel fibers (SFs) used [photo taken by authors].

Portland cement CEM I 42.5R was used according to PN-EN 197-1:2012 [40] (Górażdże Cement S.A., Chorula, Poland) (see Table 4 and Table 5). Moreover, to produce reinforced concrete substrates, steel fibers (Steelbet 50/0.75, Urban-Metal, Poland) were used (see Figure 3 and Table 6). Fiber contents of 10 kg/m^3^, 20 kg/m^3^, 30 kg/m^3^, and 50 kg/m^3^ were used.

**Table 4:** The chemical composition of the cement was measured as per PN-EN 196-2:2013-11 [41].

Composition	SiO_2_	Al_2_O_3_	Fe_2_O_3_	CaO	MgO	SO_3_	Na_2_O	K_2_O	Cl	LOI	IR
Unit (vol.%)	19.5	4.9	2.9	63.3	1.3	2.8	0.1	0.9	0.05	2.48	0.63

LOI—loss on ignition; IR—insoluble residue.

**Table 5:** The physical properties of the cement were measured according to PN-EN 196-6:2011 [42] and PN-EN 196-1:2016-07 [43].

Properties	Specific Surface Area [m^2^/kg]	Specific Gravity [kg/m^3^]	Compressive Strength [MPa]	
Material			After 2 Days	After 28 Days
Cement	3840	3060	28.0	58.0

**Table 6:** The physical and mechanical properties of the steel fibers are shown in Figure 4.

Length of Fiber L [mm]	Length l [mm]	Diameter d [mm]	Height h [mm]	Tensile Strength R_m, min_ [MPa]
50	4.0	0.75	≥3.0	100

After pouring the concrete mix with or without steel fibers, the concrete and steel fiber-reinforced substrates were stored for 28 d at a temperature of 21 ± 1 °C and a humidity above 95%. The substrates were then stored at a humidity of 60 ± 10% until testing. Screw anchors were installed in concrete substrates with or without fibers in the drill holes, and d_cut_ was nominal.

## 3. Methodology

**Figure 5:**
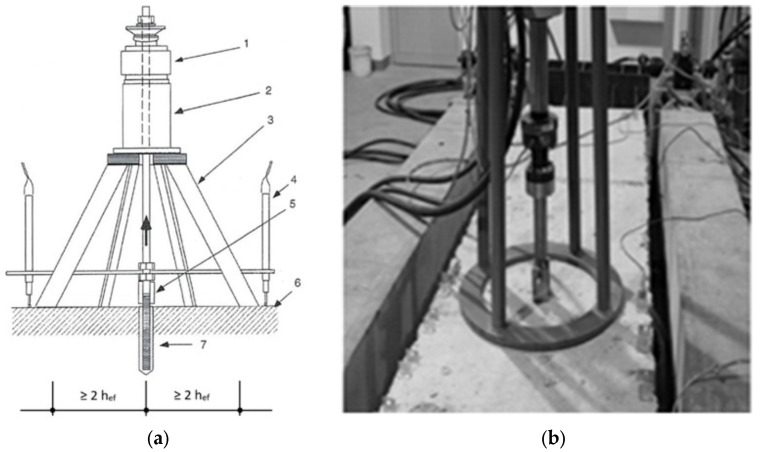
The test stand for anchor fastening. (**a**) Diagram of the test: 1—force sensor, 2—actuator, 3—support, 4—displacement sensor, 5—socket, 6—concrete, 7—screw anchor [46]. (**b**) Special test station [35].

Pull-out strength tests were carried out 90 d after the production of the concrete substrate to approximate the actual working conditions [44,45]. Load-bearing capacity tests of steel anchors installed in a C25/30 class concrete substrate without dispersed reinforcement and with the addition of steel fibers were carried out in accordance with the requirements of the EOTA [2]. Pull-out strength tests of steel anchors installed in concrete and steel fiber-reinforced concrete substrates were carried out using the following equation:

- The HBM force and displacement measurement device was compatible with the force, displacement, and pressure transducers. Data processing software from Hbm QuantumX–Catman II, operating in Windows, was used;
- C6 A force sensors (HBM, Darmstadt, Germany) were used in the range from 0 kN to 200 kN, with a force measurement resolution of 0.01 kN and a sensitivity of 2 mV/V;
- WA50 displacement sensors (HBM, Darmstadt, Germany) were used in the range of 0–50 mm, with a resolution of 0.01 mm, as shown in Figure 5.

The steel screw anchors were pulled out under the impact of static tensile forces, and the maximum destructive force was noted. The tests were performed on five samples. The load capacity (N_Ru,m_) was determined according to Formula (1).

(1) $$N_{Ru,m}=F_{Ru,m}^{t}\cdot {\left(\frac{f_{ck}}{f_{c,t}}\right)}^{0.5}$$

where

$F_{Ru,m}^{t}$—average maximum destructive force [kN];

*f_ck_*—characteristic value of compressive strength of concrete substrate [MPa];

*f_c,t_*—determined compressive strength [MPa].

**Figure 6:**
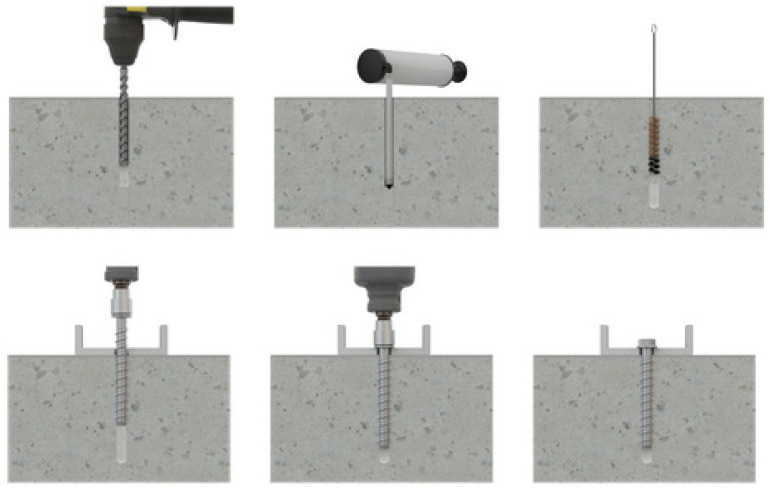
Screw anchor installation [39].

Screw anchors installed in concrete are shown in Figure 6. Installation consisted of drilling a hole into the base material of the correct diameter and depth using a drill bit that met the requirements. Dust and debris were removed from the hole using a hand pump, and compressed air or a vacuum was used to remove loose particles left from drilling. A powered impact wrench or a torque wrench that did not exceed the maximum torque was selected. An appropriately sized hex socket was attached to the wrench. A screw anchor head was mounted in the socket. The anchor was driven with an impact driver or torque wrench through the fixture and into the hole until the anchor head washer came in contact with the fixture. The anchor needed to be snug after installation.

After testing the pull-out strength of the anchors, cylindrical samples were taken from the concrete substrates with and without the addition of steel fibers according to [47,48]. The samples were tested using a MEGA 6-3000-100 compression machine (FORM + TEST, Riedlingen, Germany) with a maximum load capacity of 3000 kN. Compressive strength tests were performed on three samples with diameters of 105 mm and heights of 105 ± 1 mm per substrate according to the PN-EN 12504-1:2019-08 standard [48]. Moreover, tensile splitting tests of concrete samples with and without fibers were conducted. Tests were performed on three samples with diameters of 105 mm and heights of 210 ± 1 mm per substrate according to the PN-EN 12390-6:2011 standard [49].

## 4. Results and Discussion

### 4.1. Mechanical Properties of the Concrete Substrate

**Figure 7:**
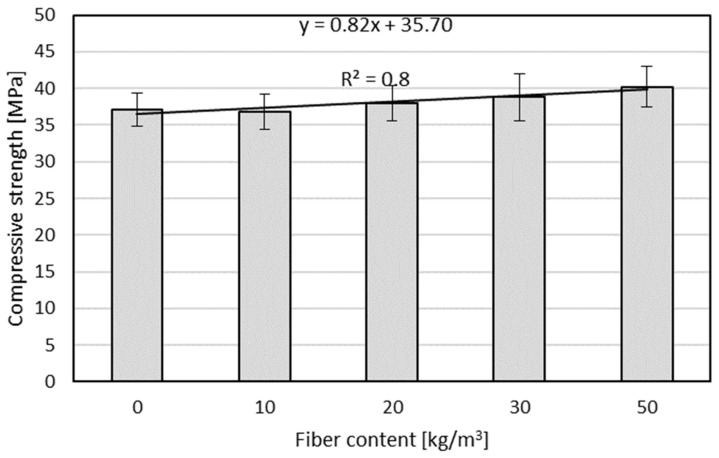
Ninety-day compressive strengths of concrete substrates with and without the addition of steel fibers.

**Figure 8:**
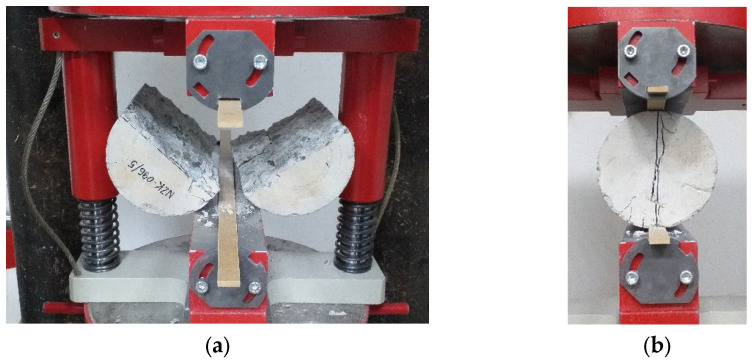
Images of the destruction of samples taken from the concrete substrates without (**a**) and with the addition of 10% steel fibers (**b**) after the tensile splitting strength test.

Table 7 and Figure 7 present the results of the mechanical properties of the concrete substrate of the C25/30 class without fibers (base sample) and with the addition of steel fibers. Although the purpose of the fibers was not to increase the compressive strength [28,50], as the fiber content increased, the compressive strength increased (see Figure 7). The standard deviation of the compressive strength was approximately 1 MPa. Moreover, the effect of the fiber reinforcement of the concrete is visible in the failure image from the tensile splitting strength test (Figure 8).

### 4.2. The Results of the Pull-Out Test

The results of the pull-out tests of the screw anchors installed in the concrete substrates of the C25/30 class without fibers and with the addition of fibers are listed in Table 8. An increase in the average maximum destructive force was observed depending on the fiber content in the concrete mixture of the substrate.

**Figure 9:**
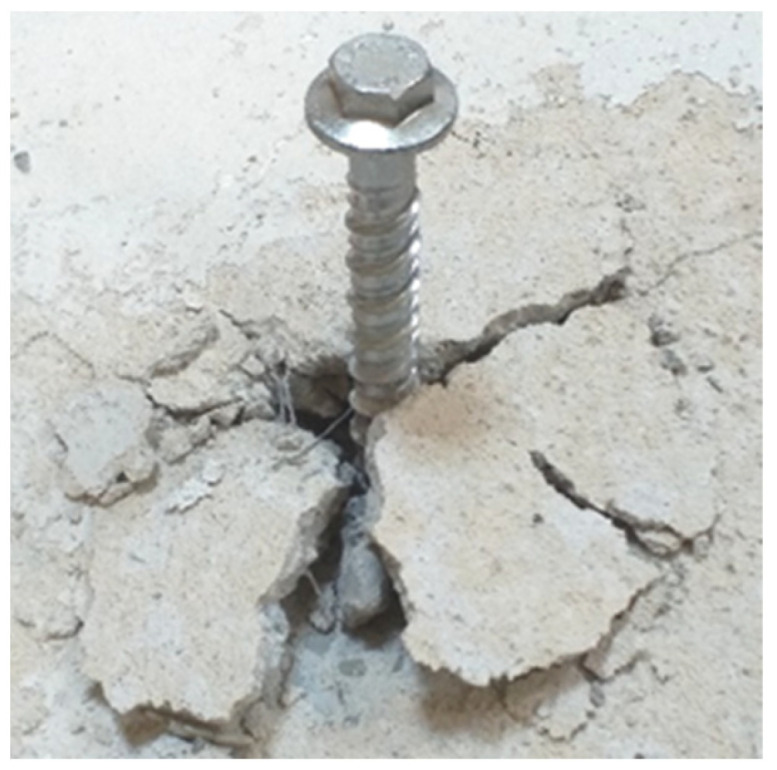
The characteristic failure mode of the connection was observed with the test screw anchors in fiber-reinforced concrete.

The greatest effect of fiber addition was observed for an effective depth of 45 mm, followed by an effective depth of 79 mm. Figure 9 presents the characteristic failure mode of screw anchors in fiber-reinforced concrete observed in the pull-out tests. This was different from that observed for unreinforced substrates [22,51,52].

**Figure 10:**
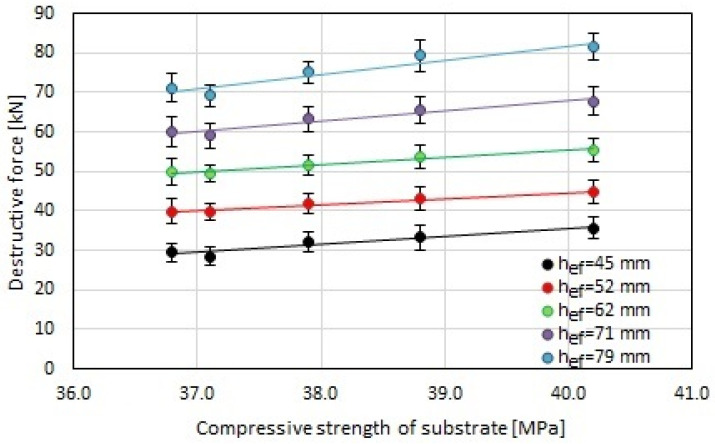
The correlation between the average destructive force of the screw anchors and the 90-day compressive strength of the concrete substrate with and without the addition of steel fibers, which depends on the effective depth (h_ef_).

In the first case, the maximum destructive force increased by approximately 3%, 13%, 17%, and 25% compared to the reference sample for steel fiber contents of 10, 20, 30, and 50 kg/m^3^, respectively. In the second case, the destructive force increased by approximately 3%, 8%, 15%, and 21%, respectively. For the remaining effective depths, the increases in the maximum destructive force with the steel fiber content were not spectacular and amounted to approximately 1–2%, 5–7%, 9%, and 12–15% compared to the reference sample for steel fiber contents of 10, 20, 30, and 50 kg/m^3^, respectively. Therefore, a significant improvement in the value of the maximum destructive force was obtained with the steel fiber content in the concrete substrate. This may be related to the increase in the strength of the steel fiber-reinforced substrate, as shown in Figure 7. Figure 10 presents a linear correlation between the average destructive force of the screw anchors and the compressive strength of the concrete substrate with and without the addition of steel fibers, which depends on the effective depth.

**Figure 11:**
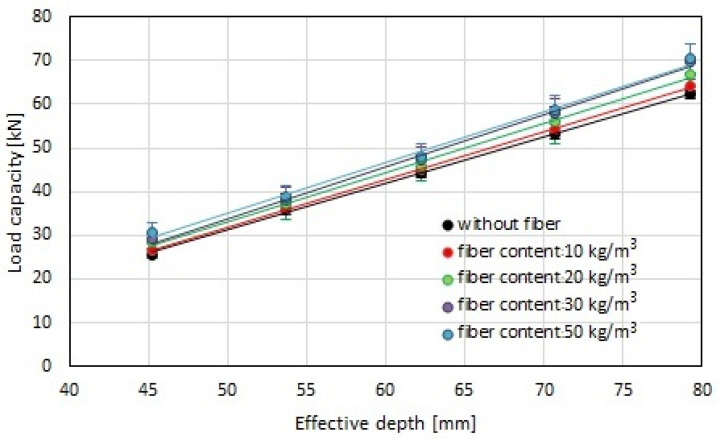
The correlation between the load capacity and the effective depth, h_ef_, depends on the fiber content in the concrete substrate.

In the next step, the load capacity was determined according to Equation (1). The results are shown in Figure 11. A linear correlation between the load capacity of screw anchors installed in the fiber-reinforced concrete substrate and the effective depth was determined, which was consistent with previous test results, for example, for expansion connectors [35,46]. However, it can be observed that the effect of the fiber content on the load capacity for a given effective depth was slight. For higher effective depths, a greater effect of the steel fiber content on the load capacity of the screwed anchor was observed.

**Figure 12:**
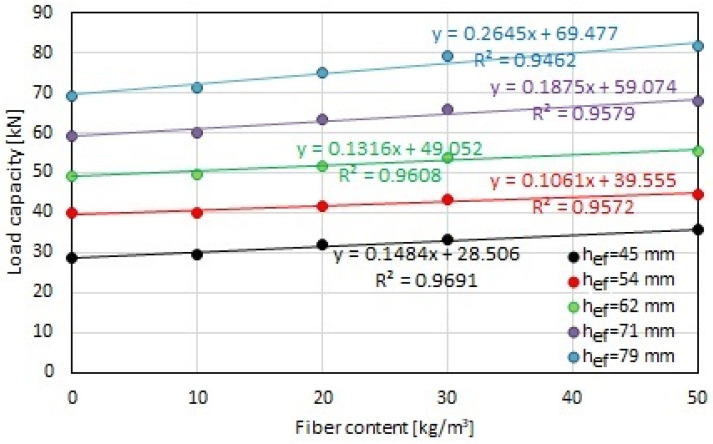
Correlation between load capacity and fiber content in concrete substrate depending on effective depth (h_ef_).

Figure 12 shows the correlation between the load capacity and the steel fiber content depending on the effective depth. It is possible to notice a smaller effect of the fiber content on the load capacity of screw anchors for effective depths of 54 mm and 62 mm, which is visible in the flattening of the curve. At higher effective depths, a greater impact of the steel fiber content on the load capacity of the screwed anchor was observed. The difference in load capacity was up to 20% for anchors with a length of 120 mm (anchor 1), for which the effective depth was 45 mm, and up to 13% for anchors with a length of 140 mm (anchor 2), for which the effective depth was 79 mm. However, for effective depths of 54 mm (anchor 1) and 62 mm (anchor 2), this difference was approximately 8%, and for an effective depth of 71 mm (anchor 2), it was 10%.

Figure 12 presents a linear correlation for all analyzed effective depths. Pearson coefficients above 0.9 were obtained for all correlations, which indicates a good fit for the curves.

The fasteners of the mechanical anchors were designed in accordance with the Concrete Capacity Method specified in the PN-EN 1992-4 standard [3]. The characteristic values of the load capacity of the anchors installed in the concrete substrate (*N_Rk,p_*) can be calculated according to Formula (2).

(2) $$N_{Rk,p}=k_{ucr,N}\cdot h_{ef}^{1.5}\cdot f_{ck}^{0.5}$$

where

*k_ucr,N_*—correction factor [-] according to Eurocode [3];

*h_ef_*—effective depth of fasting screw anchor in the concrete substrate [mm];

*f_ck_*—characteristic value of compressive strength of concrete substrate [MPa].

Based on the obtained results of the pull-out test for the screw anchors installed in the concrete substrates (with and without the addition of steel fiber), the characteristic value of the load capacity (*N_Rk,p_*) for each case can be determined according to Formula (3).

(3) $$N_{Rk,p}=N_{Ru,m}\cdot \left(1-k_{s}\cdot \nu_{F}\right)$$

where

*k_s_*—coefficient for a tolerance level of 95% (defectiveness level of 5%) and a confidence level of 90%, depending on the sample size;

$\nu_{F}$—coefficient of variation calculated according to Formula (4).

(4) $$\nu_{F}=\frac{s}{F_{Ru,m}}\cdot 100$$

where

*s*—standard deviation.

**Figure 13:**
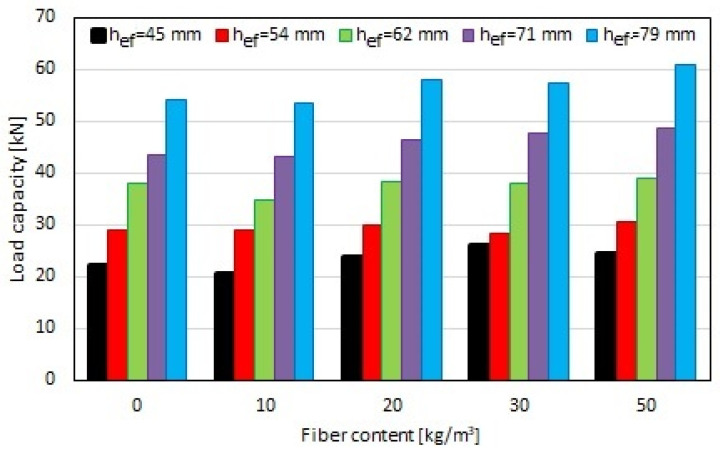
Summary of the results of the characteristic value of the load capacity of screw anchors in fiber-reinforced concrete substrates depending on the effective depth (*h_ef_*).

The results for the characteristic value of the load capacity of the screw anchors in the concrete substrates with and without the addition of steel fiber, determined based on Formula (3), are presented in Figure 13.

Based on Formula (2) and the characteristic value of the load capacity (*N_Rk,p_*) calculated from the results obtained in laboratory tests for non-cracked concrete substrates with the addition of steel fibers, the *k_ucr,N_* coefficient was determined. The results of the *k_ucr,N_* coefficient for different steel fiber contents are listed in Table 9.

The lowest value of *k_ucr,N_*, with a coefficient of 13.10, was obtained. Therefore, this value of the *k_ucr,N_* coefficient is recommended for Steelbet 50/0.75 fibers.

The lower *k_ucr,N_* coefficient from Table 9 can be taken into account when assessing the safety of a building structure. This value of the *k_ucr,N_* coefficient is recommended as a reference value for determining the characteristic value of the load capacity of screw anchors installed in a non-cracked concrete substrate of class C25/30 with the addition of Steelbet 50/0.75 fibers, based on Formula (2).

## 5. Summary and Conclusions

This study aimed to assess the possibility of using screw anchors installed in concrete substrates of class C25/30 without fibers and with the addition of steel fibers (10 kg/m^3^, 20 kg/m^3^, 30 kg/m^3^, and 50 kg/m^3^). Different values of effective depth were used (45, 54, 62, 71, and 79 mm). The following conclusions can be drawn based on the results of this experimental study:

1. Steel fibers affect the destructive force of screw anchors, increasing the pull-out strength for each effective depth.
2. A significant improvement in the value of the maximum destructive force was obtained for the concrete substrate with the addition of steel fiber.
3. With increasing effective depth (effective depth (*h_ef_*) = 45 mm–79 mm), the destructive force of screw anchors increases; this correlation is linear for screw anchors installed in concrete substrates without fiber and with the addition of fiber.
4. To calculate the characteristic value of the load capacity of screw anchors in the fiber-reinforced concrete substrate with the addition of steel fiber, a *k_ucr,N_* coefficient of 13.10 can be used. This was the lowest value obtained in this study for the load capacities of screw anchors in the fiber-reinforced concrete substrate.

This study is part of a wider research project aimed at assessing the actual load capacities of fasteners in steel fiber-reinforced concrete substrates. A novelty is the testing of screws screwed into steel fiber-reinforced substrates. So far, tests have been conducted for expansion and bonded anchors. As this research continues, testing of other types and contents of steel fibers is planned.

## Figures and Tables

**Figure 2:**
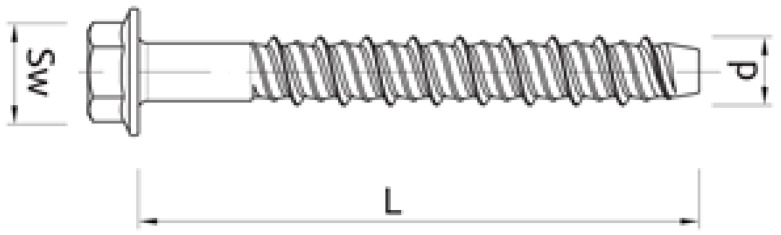
Geometry of steel screw anchors used [39].

**Figure 4:**
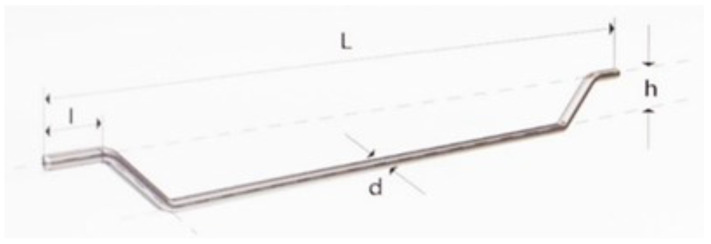
Geometry of the steel fibers (SFs) used.

**Table 2:** Installation parameters of the screw anchors used.

No.	Type of Screw Anchor	Drill Diameter d_0_ [mm]	Drill Depth h_1_ [mm]	Embedment Depth h_nom_ [mm]	Effective Depth h_ef_ [mm]
1	TFE14120	14	75	65	45
			85	75	54
2	TFE14140		95	85	62
			105	95	71
			115	105	79

**Table 3:** Composite concrete mix [1 m^3^].

Type of Composite	Composite	Density [kg/m^3^]	Content [kg]
Aggregate	Sand 0/2	2660	747
Aggregate	Gravel 2/8	2640	362
Aggregate	Gravel 8/16	2630	811
Portland cement	CEM I 42.5R	3060	292
Water	Tap water	998	153
Admixture	MasterPozzolith BV 18C (0.40% of cement mass)	1095	1.16
Admixture	Sikament 400/30 (0.70% of cement mass)	1080	2.04

**Table 7:** Strength results of the concrete substrate.

No.	Fiber Content [kg/m^3^]	Average Compressive Strength	Average Tensile Splitting Strength
		*f_c,t_* [MPa]	*f_c,t_* [MPa]
1	Reference sample	37.1 ± 0.8	3.15 ± 0.12
2	10	36.8 ± 1.0	3.18 ± 0.14
3	20	37.9 ± 1.1	3.34 ± 0.21
4	30	38.8 ± 1.2	3.58 ± 0.18
5	50	40.2 ± 1.3	4.38 ± 0.21

**Table 8:** Results of pull-out test depending on effective depth.

No.	Fiber Content [kg/m^3^]	Average Maximum Destructive Force $F_{Ru,m}^{t}$ [kN]				
		Effective Depth *h_ef_* [mm]				
		45	52	62	71	79
1	Reference sample	28.45 ± 1.12	39.89 ± 2.31	49.34 ± 2.10	59.10 ± 3.23	69.23 ± 2.67
2	10	29.39 ± 1.97	39.94 ± 2.37	49.72 ± 3.35	60.11 ± 3.67	71.24 ± 3.60
3	20	32.17 ± 1.61	41.77 ± 2.41	51.60 ± 2.55	63.21 ± 3.24	75.07 ± 2.88
4	30	33.25 ± 1.10	43.12 ± 3.21	53.69 ± 3.12	65.73 ± 3.39	79.34 ± 4.17
5	50	35.59 ± 2.16	44.73 ± 2.77	55.38 ± 3.08	67.84 ± 3.41	81.60 ± 3.34

**Table 9:** Designated values of the *k_ucr,N_* coefficient.

	Fiber Content [kg/m^3^]			
	10	20	30	50
*k_ucr,N_* coefficient	13.10	14.32	14.45	14.77

## Data Availability

Data are contained within the article.
